# Recent Catalytic Applications of Ferrocene and Ferrocenium Cations in the Syntheses of Organic Compounds

**DOI:** 10.3390/molecules29235544

**Published:** 2024-11-23

**Authors:** Eike B. Bauer

**Affiliations:** Department of Chemistry and Biochemistry, University of Missouri—St. Louis, One University Boulevard, St. Louis, MO 63121, USA; bauere@umsl.edu; Tel.: +1-(314)-516-5311; Fax: +1-(314)-516-5342

**Keywords:** ferrocenium, oxidation, coupling reactions, polymerization reactions, Lewis acid catalysis, redox-switchable catalysts

## Abstract

Ferrocene and its oxidized counterpart, the ferrocenium cation, represent a fascinating class of organometallic compounds with broad utility across various fields, including organic synthesis, pharmaceuticals, and materials science. Over the years, ferrocene, ferrocenium cations, and their derivatives have also gained prominence for their versatility in catalytic processes. This review article offers an overview of the research of the last decade into ferrocene- and ferrocenium-based catalysis. Key developments are highlighted in catalytic oxidation, cross-coupling, polymerization reactions, and redox-switchable catalysis, as well as the application of ferrocenium cations as Lewis acid catalysts.

## 1. Introduction

Ferrocene, first synthesized in 1951, marked a groundbreaking discovery in organometallic chemistry due to its unique “sandwich” structure, where an iron atom is symmetrically positioned between two cyclopentadienyl rings [1,2]. Its discovery significantly advanced the understanding of bonding in organometallic compounds, which assisted in their development for various applications in catalysis and materials science.

**Scheme 1:**
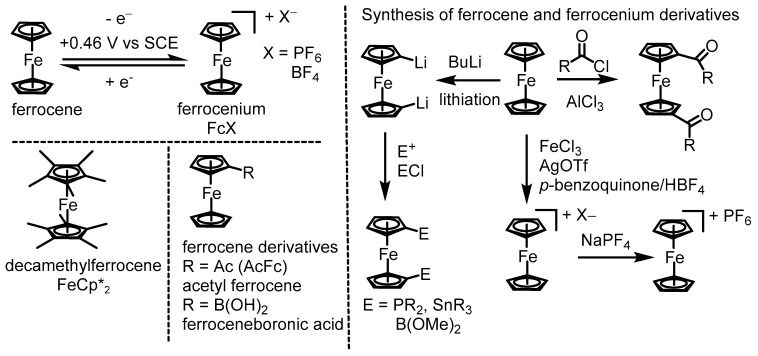
Derivatives, naming and synthesis of ferrocene derivatives.

Ferrocene itself (Fc denoted in Schemes throughout the text) is a very stable compound [2]. For instance, it has been used as a platform for synthesizing phosphorus-based ligands [3,4,5,6], including chiral variants suitable for enantioselective catalysis [7,8,9]. Ferrocene derivatives have also been researched as bioactive compounds [10,11,12] or in the field of material science [13]. The oxidation of ferrocene is straightforward and leads to the ferrocenium cation. The reversible, one-electron ferrocene/ferrocenium redox couple, which is well behaved, serves as a standard in electrochemistry (Scheme 1, top) [14]. The ferrocenium cation (denoted as Fc^+^ in this text) is significantly more reactive than ferrocene, though ferrocene itself has also been used as a catalyst. The ferrocenium cation has found applications both stoichiometrically and catalytically in various synthetic processes [15]. Ferrocene can be electronically and sterically tuned through the cyclopentadienyl ligands (see Scheme 1, bottom, including naming used throughout the text) [16,17], and therefore, the chemical properties of ferroceniums cations (which are accessible through oxidation from their respective ferrocenyl precursors) can be modified. The ferrocenium cation itself serves as a Lewis acid, which has been taken advantage of in catalytic applications. The fine-tuning of the Lewis acidity is possible though substituents on the ring system. For example, acetyl ferrocene (AcFc, Scheme 1) has an oxidation potential 0.27 V higher than ferrocene, while decamethyl ferrocene (FeCp*_2_, Scheme 1) has an oxidation potential 0.59 V lower than ferrocene [14]. The reversible redox behavior of ferrocene allows for participation in catalytic cycles involving electron transfer. Their ability to act as both Lewis acids and electron mediators has made ferrocenium cations particularly attractive in areas such as cross-coupling reactions or redox-switchable catalytic applications.

The synthesis of ferrocenes substituted at their Cp rings is relatively straightforward; a few approaches are shown in Scheme 1 (right) [18,19]. The Cp ring systems exhibit aromatic-like chemical properties, enabling them to undergo electrophilic aromatic substitution reactions [20,21]. The lithiation of Cp rings produces lithiated intermediates, which can then react with various electrophiles (E^+^) to introduce desired substituents (Scheme 1, right) [22]. Additionally, ferrocene and its derivatives can be readily oxidized using oxidants such as FeCl_3_ [23], silver triflate [24], or *p*-benzoquinone [25]. Counterion exchange can be achieved with sodium or ammonium salts of the general formula NaX or NH_4_X [23,26].

Iron catalysis [27] offers significant advantages in organic synthesis due to its non-toxicity, environmental friendliness, low cost, and abundance [28,29]. As a non-toxic metal, iron presents a safer alternative to more hazardous transition metals like palladium or platinum, which are often used in catalytic processes but are substantially more toxic and much more expensive [30]. Its abundant availability in the Earth’s crust makes iron not only cost-effective but also sustainable for large-scale industrial applications. As such, iron-based catalyst systems follow the principles of green chemistry [31]: they overall minimize environmental impact and are non-toxic, sustainable, and economically viable [32]. Therefore, ferrocenium-based catalysis is an attractive area for developing greener, more efficient synthetic methods.

The current state of ferrocenium catalysis provides valuable new tools for synthetic organic chemists [15]. While early research highlighted the fundamental catalytic roles of ferrocenium cations, recent advancements have focused on fine-tuning their activity through modifications of the cyclopentadienyl rings [17,33] and incorporating ferrocenium units with other transition metals [34]. This review aims to evaluate the scope and limitations of these developments by exploring key examples from the past decade of research. Additionally, earlier examples will be included where relevant to illustrate the concepts. The catalytic activity of ferrocene or ferrocenium cations in oxidation reactions, coupling reactions, Lewis acid catalysis, polymerization reactions, and redox-switchable catalysts will be discussed. Finally, this article will offer an outlook on future directions and potential innovations in ferrocenium-based catalysis.

## 2. Ferrocenium-Catalyzed Oxidation Reactions

Iron-catalyzed oxidation reactions are well established [35]. Ferrocenium-catalyzed oxidation reactions attracted attention early on. As the oxidized form of ferrocene, ferrocenium cations Fc^+^ can act as mild, one-electron oxidants, and there are numerous applications of oxidation reactions employing stoichiometric amounts of the ferrocenium cation [14,36]. However, the reversible redox behavior of the ferrocene/ferrocenium redox couple allows, also, for catalytic applications. In most cases, the ferrocenium ion serves as an electron shuttle, which is reoxidized by a stoichiometric oxidant after it oxidizes a substrate molecule, completing the catalytic turnover. This concept is also taken advantage of in electrosynthesis, and this topic has recently been reviewed [37]. Ferrocenium cations also react with peroxide oxidants to create oxygen-centered radicals, inducing Fenton-type chemistry [38]. Ferrocenium catalyzed oxidation reactions offer an environmentally friendly alternative to traditional oxidants, which are often based on metals such as chromium or osmium. These features make ferrocenium-catalyzed oxidation reactions a promising area for developing more sustainable oxidation methodologies.

**Scheme 2:**
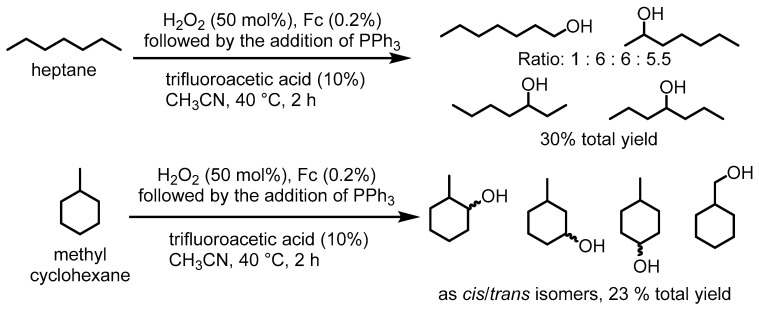
Ferrocene-catalyzed oxidation of alkanes [39].

The principle of oxidations involving ferrocene was employed by Shul’pin in the ferrocene-catalyzed oxidation of alkanes and benzene with hydrogen peroxide as the oxidant [39]. Heptane, benzene, and methylcyclohexane were oxidized to the corresponding alcohols when treated for two hours at 40 °C with ferrocene, with H_2_O_2_ as the oxidant, and pyrazine-2-carboxylic acid (PCA) or trifluoroacetic acid (TFA) as the cocatalyst in turnover numbers around 1200 (Scheme 2). As can be seen, mixtures of isomers were obtained. Mechanistic investigations (as well as a look at the product distribution) revealed that the reaction proceeded through ·OH radicals, which were generated by the ferrocene. Additionally, labeling experiments have shown that atmospheric oxygen was incorporated into the products, providing further evidence for the presence of free radicals. Although the synthetic applications of this system are currently limited, it offers valuable mechanistic insights into ferrocene-catalyzed oxidation reactions. Its simplicity may hold promise for potential use in large-scale alcohol synthesis.

**Scheme 3:**
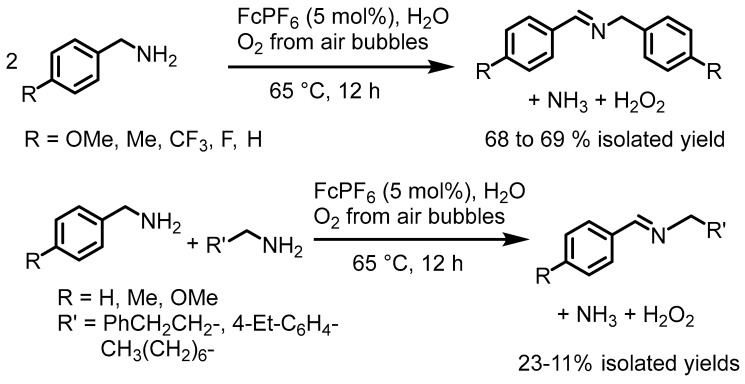
Oxidation of benzylic amines to imines [40].

Elias reported a ferrocenium-promoted method for the chemoselective oxidation of benzylic amines to afford the corresponding imines, achieving yields ranging from 53% to 93% (Scheme 3) [40]. The reactions worked by employing just 5 mol % of ferrocenium hexafluorophosphate; air was the terminal oxidant and water served as the solvent. Therefore, the reaction followed the principles for Green Chemistry in several ways. Cross-couplings were also possible (Scheme 3, bottom). Again, a radical mechanism was suggested by the authors, where the ferrocenium cation generated a benzyl amine radical, which, through the reaction with oxygen from the air, generated benzaldehyde in solution. The benzaldehyde condensed with aniline to the imine product. The ferrocene generated during the reaction was re-oxidized to enter another catalytic cycle. The isolated yields of the cross-couplings were not very high, but the simplicity of the reaction and the affordable reagents may make it an interesting method for large-scale applications.

Contaminants in wastewater pose a significant environmental challenge, and the removal of these pollutants is crucial for protecting ecosystems, improving water quality, and ensuring public health. The oxidative degradation of pollutants is a widely researched approach aimed at breaking down harmful compounds in the environment, offering an effective method to reduce contamination [41,42].

**Scheme 4:**
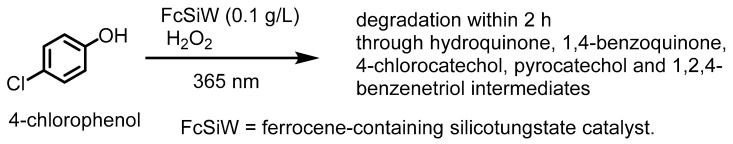
Degradation of 4-chlorophenol using a heterogeneous catalyst [43].

Li described a ferrocene containing silicotungstate as a heterogeneous photocatalyst to induce Fenton-type [38] chemistry (Scheme 4) [43]. This catalyst was proven to degrade 4-chlorophenol—a model compound for pollutants—within 2 h in the presence of H_2_O_2_ and under irradiation. Hydroquinone, 1,4-benzoquinone, 4-chlorocatechol, pyrocatechol, and 1,2,4-benzenetriol were determined as intermediates in the reaction, which proceeded though hydroxyl radicals generated with the aid of the ferrocene. 

**Scheme 5:**
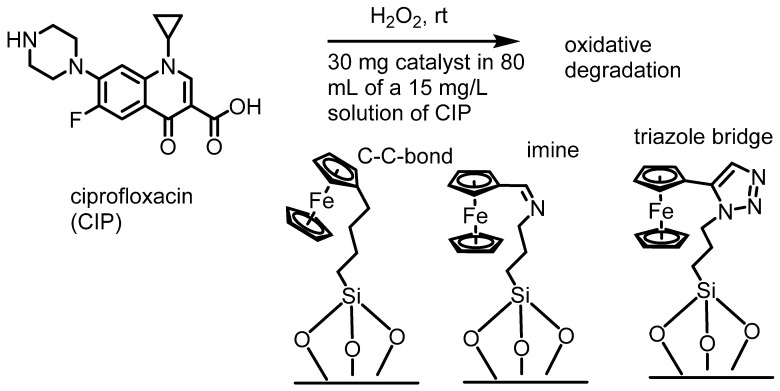
Oxidative ciprofloxacin decomposition [44].

Pharmaceuticals, like the commonly detected antibiotic ciprofloxacin, can also contribute to wastewater contamination. Ziolek reported a heterogeneous catalyst system for the oxidative degradation of ciprofloxacin in wastewater (Scheme 5) [44]. In this system, ferrocene was anchored on the surface of mesoporous silica SBA-15 through Schiff base (imine) formation, Friedel–Crafts alkylation, and azide–alkyne click reactions. The resulting ferrocene-containing solid material was used to degrade ciprofloxacin in the presence of H_2_O_2_. A correlation was found between the ferrocene content in the solid catalyst and its efficiency, with the azide chemistry proving to be the most effective method for tethering ferrocene, resulting in the highest ferrocene content. The greater the ferrocene content, the higher the catalytic activity in degrading ciprofloxacin. The authors suggest that ferrocene, upon oxidation to ferrocenium, generates hydroxyl radicals in Fenton-type chemistry [38], which are responsible for the degradation of ciprofloxacin.

Ferrocenes and its derivatives also found applications in oxidation chemistry as burning rate catalysts for solid-state propellants. These catalysts were added to propellants to enhance or regulate the combustion rate, with applications such as rocket propulsion. Ferrocene-containing polymers have been studied for this purpose [45,46,47]. Ferrocene-based metal–organic frameworks have also been identified as oxygen evolution catalysts [48,49], with potential applications in water splitting and hydrogen production. 

As demonstrated in the examples above, ferrocenium cations catalyze various oxidation reactions, using H_2_O_2_ or oxygen as the terminal oxidant. The authors of these studies commonly propose Fenton chemistry as the mechanism behind H_2_O_2_-based oxidations, where hydroxyl radicals (·OH), powerful yet non-selective oxidants, are generated. While Fenton chemistry is effective, the high reactivity of hydroxyl radicals limits their use in synthetic organic chemistry, though they appear to be well suited for the oxidative degradation of pollutants. Consequently, Fenton chemistry has been investigated for wastewater treatment before [42]. Future research may focus on ferrocenium-catalyzed oxidation reactions that involve less reactive species. A simple approach would be to use *t*BuOOH instead of H_2_O_2_, as has been previously applied in oxidation reactions [50].

## 3. Ferrocenium-Catalyzed Coupling Reactions

A coupling reaction is a process in which two fragments are joined together to form a single, larger molecule, typically with the aid of a catalyst [51]. These reactions are commonly used in organic synthesis to form carbon–carbon or carbon–heteroatom bonds and are playing a crucial role in the creation of complex molecules such as pharmaceuticals [52]. Ferrocene-based ligands have been employed extensively in the cross-coupling reactions catalyzed by a number of transition metals [7,53]. Ferrocenium cations themselves have also been employed in coupling reactions.

**Scheme 6:**
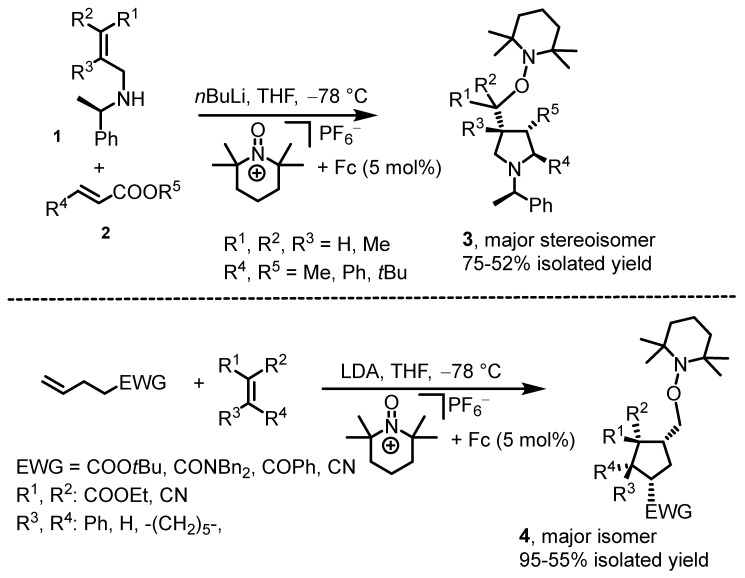
Single-electron transfer-induced tandem anion radical reactions [54].

Jahn applied the concept of oxidative catalysis to facilitate single-electron transfer-induced tandem anion radical reactions to access highly functionalized and substituted pyrrolidines and cyclopentanes [54]. In the catalytic tandem aza-Michael addition followed by radical cyclization and oxygenation (Scheme 6, top), the process began with a Michael addition from **1** to **2**, after which the resulting adduct was oxidized by a ferrocenium cation, generating a radical and ferrocene. The radical underwent cyclization, and the cyclic radical was then trapped with TEMPO in a final step to afford product **3**. TEMPO was produced from *N*-oxopiperidinium hexafluorophosphate, which was added as a stoichiometric oxidant to regenerate the ferrocenium catalyst. A “regular” tandem Michael addition/cyclization/oxygenation sequence was also possible under these conditions to afford substituted cyclopentanes **4** (Scheme 6, bottom). These reactions produced one of the several possible diastereomers as the major product, and the selectivity was obviously driven by product stability, as analyzed by X-ray crystallography of the products. The reaction is atom economic, and, according to the authors, the first of this kind.

**Scheme 7:**
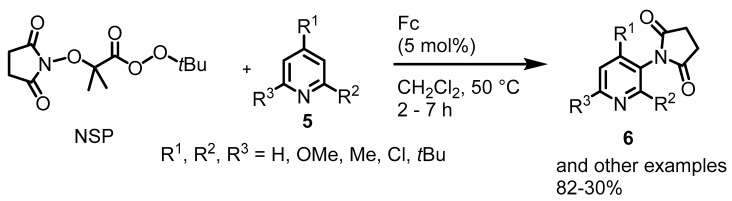
Direct C-H imidation of heteroarenes [55].

Baran published a ferrocene-catalyzed, direct C–H imidation of arenes and heteroarenes (Scheme 7) [55]. In that system, *N*-succinimidyl perester (NSP) serves as an imide source, which is coupled to a heteroaromatic ring **5**. The resulting succinimide products **6** can be deprotected in situ to obtain the corresponding amines. The regioselectivity of the imidation mirrors that of electrophilic aromatic substitution: electron-donating groups like alkoxy and alkyl direct substitution to the *ortho*/*para* positions. Mechanistically, single-electron transfer from ferrocene to NSP triggers O–O bond cleavage, producing an imidyl radical, CO_2_, acetone, and ferrocenium *t*butoxide. The imidyl radical reacts with the aromatic substrate to form a radical adduct, which undergoes one-electron oxidation and deprotonation to yield the product, while regenerating ferrocene. The ferrocene serves as electron shuttle in the reaction.

**Scheme 8:**
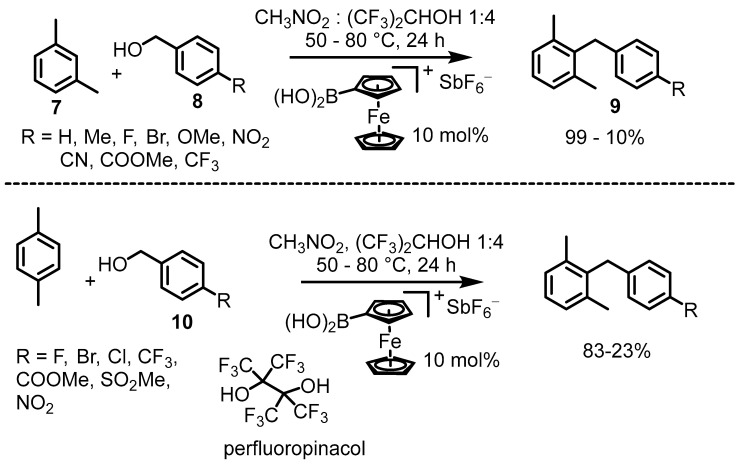
Friedel-Crafts reactions with benzylic alcohols [56,57].

Hall described the application of the ferroceniumboronic acid hexafluoroantimonate salt as a catalyst in Friedel–Crafts reactions between slightly activated and neutral arenes **7** and primary and secondary benzylic alcohols **8** (Scheme 8, top) [56]. The reaction afforded unsymmetrical diaryl arenes **9** in up to a 99% yield and isomeric ratios around 80:20. The ferroceniumboronic acid exhibited unique catalytic activity, because it was more reactive compared to FeCl_3_ and Bi(OTf)_3_ and had a broader substrate scope. Interestingly, both the ferrocenium cation and unoxidized ferroceneboronic acid alone led to significantly lower yields in the reaction. The ferroceniumboronic acid did not just act as a Brønsted acid, because boronic acids of similar acidity are not catalytically active. It was the combination of the free boronic acid with the ferrocenium core that formed a highly active catalyst. The authors assumed that the ferroceniumboronic acid assisted with the formation of a carbocation from the benzylic alcohol by forming a boronate. The carbocation was attacked by the aryl ring and the proton liberated through this process regenerated the boronic acid.

The Friedel–Crafts reaction in Scheme 8 (top) did not work well with deactivated benzylic alcohols. However, the authors discovered that adding perfluoropinacol produced a more active catalyst, enabling the activation of deactivated benzylic alcohols **10** for the reaction (Scheme 8, bottom) [57]. They proposed that perfluoropinacol forms a five-membered boronic ester with the boronic acid in situ (as detected by ESI-MS), creating a more electrophilic, Lewis acidic boron center.

**Scheme 9:**
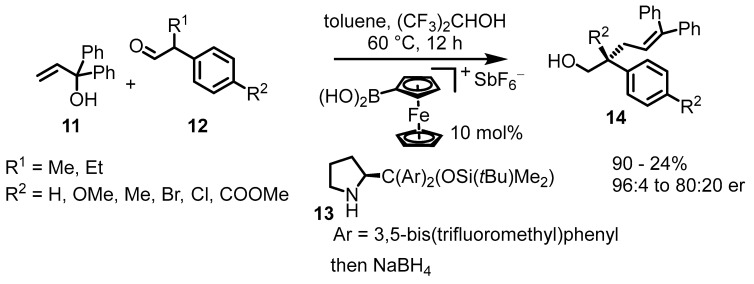
Enantioselective alkylation of branched aldehydes with allylic alcohols [58].

The same authors used ferrocenium boronic acid in combination with chiral amine **13** for the enantioselective alkylation of branched aldehydes **12** with allylic alcohols **11** (Scheme 9) [58]. This reaction yielded chiral compounds **14** with quaternary stereocenters. Similarly to the mechanism in Scheme 8, the authors proposed that the allylic alcohol **11** forms a carbocation, which then reacts with a chiral enamine generated from aldehyde **12** and chiral amine **13**. The attempts using 2,3,4,5-F_4_HC_6_B(OH)_2_ and ferrocenium hexafluoroantimonate (FcSbF_6_) resulted in lower yields, highlighting the essential role of the ferrocenium boronic acid catalyst in the reaction.

**Scheme 10:**
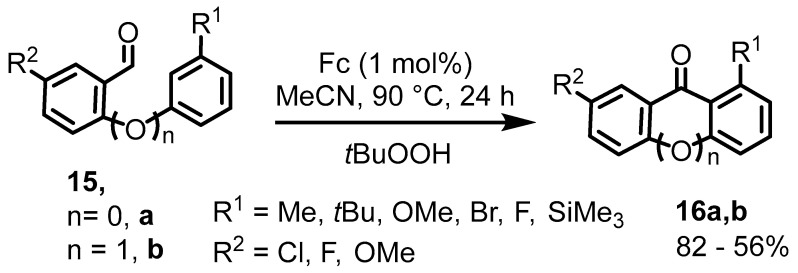
Cross-dehydrogenative coupling to access fluorenones [50].

Cross dehydrogenative coupling (CDC) reactions via base-promoted homolytic aromatic substitutions were reported by Studer [50]. These reactions produced fluorenones **16a** and, in slightly lower yields, xanthones **16b**, starting from *ortho*-formyl biphenyls **15a** or *ortho*-formyl biphenyl ethers **15b** (Scheme 10). The oxidant *t*BuOOH was used in the process in the presence of ferrocene (0.1 to 1 mol%) as the catalyst. Under these conditions, *t*BuO· radicals formed, which initiated a radical chain by abstracting a hydrogen from the aldehyde group to afford an acyl radical which attacked the neighboring aromatic ring.

**Scheme 11:**
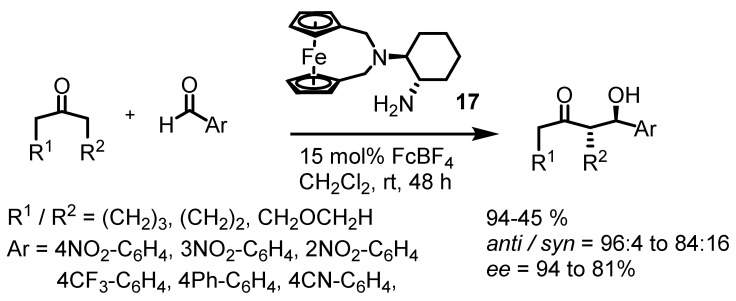
Enantioselective aldol reaction [59].

Cui, Luo, and Wo provided an example of asymmetric aminocatalysis using a chiral ferrocenophane catalyst **17** (Scheme 11) [59]. When oxidized with ferrocenium tetrafluoroborate (FcBF_4_) in situ, the catalyst was active in asymmetric aldol reactions at room temperature. Other oxidants gave lower *syn*/*anti* diastereoselectivities. The catalyst **17** could be recovered via precipitation and reused several times with little loss of activity. The oxidized ferrocenophanium ion was proposed to act as a Lewis acid, coordinating the aldehyde to the iron center, thereby contributing to the stereocontrol of the reaction.

As can be seen from the examples above, ferrocenium-catalyzed cross-coupling reactions are well developed and have proven highly effective in synthesizing products with significant structural complexity. Some studies highlighted the superior performance of ferrocenium catalysts compared to other alternatives, underscoring its value as a catalyst in advanced synthetic chemistry [56,58]. Advancing the field with respect to the type of cross coupling reactions and widening the substrate scope remains an ongoing target.

## 4. Various Reactions Where the Ferrocenium Serves as a Lewis Acid Catalyst

In the examples above, the ferrocenium cation served mainly as an electron shuttle or as an activator for Fenton chemistry. There were a variety of reactions where the ferrocenium cation served as a Lewis acid. 

**Scheme 12:**
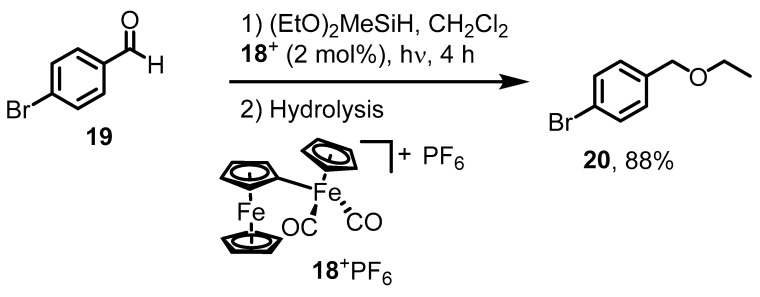
Reductive etherification of aldehydes [60].

For example, Argouarch, Humphrey, and Paul reported the employment of the binuclear ferrocenium cation **18**^+^ in the reductive etherification of aldehyde **19** to obtain the ether product **20** in an 88% yield (Scheme 12) [60]. A silane served as the reductant. In its oxidized form, the charge in **18**^+^ was delocalized and the complex was remarkably stable. Consequently, the oxidation potential to obtain **18**^+^ from its reduced form **18** was low. The neutral complex **18** was also catalytically active in the reductive etherification.

**Scheme 13:**
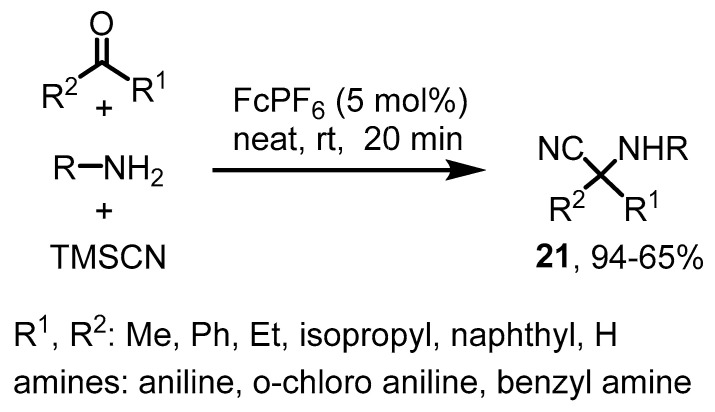
FcPF_6_-catalyzed Strecker reaction [61].

Khan reported a FcPF_6_-catalyzed three-component Strecker reaction between aldehydes, amines, and TMS-CN (Scheme 13) [61]. At a catalyst load of 5 mol%, the corresponding α-aminonitriles **21** were obtained in an up to 94% yield after only 20 min at room temperature without a solvent involved. In this case, the authors assumed the Lewis acidic ferrocenium cation polarized the C=N bond of the imine, which formed as an intermediate between the amine and the aldehyde. The polarized imine was then attacked by the cyanide nucleophile.

**Scheme 14:**
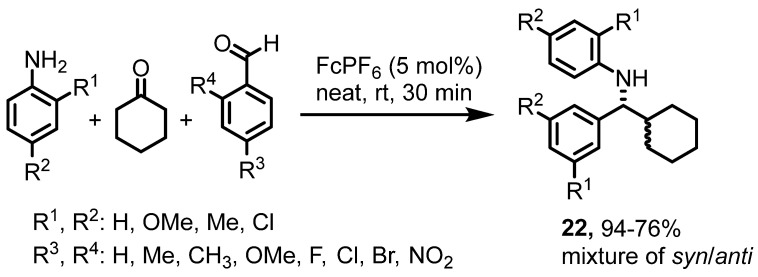
Mannich reaction [62].

Kureshy reported a comparable three-component reaction between the aldehydes, ketones, and amines, known as the Mannich reaction (Scheme 14) [62]. The ß-amino-ketone products **22** were obtained again at a catalyst load of 5 mol% after 30 min at room temperature under neat conditions as *syn*/*anti* mixtures. The *anti*-isomer was obtained in excess. Again, the authors showed via mechanistic experiments that, first, an imine is formed, which is then attacked by the enol form of the ketone. While not proven or mentioned by the authors, it is reasonable to assume that FcPF_6_ serves as a Lewis acid that helps to polarize the imine and with tautomerizing the cyclohexyl ketone to the enol.

**Scheme 15:**
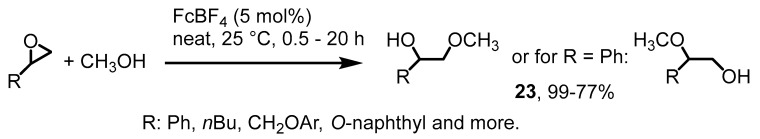
Alcoholysis of Epoxides [63].

Ferrocenium tetrafluoroborate was employed as a catalyst by Singh in the ring-opening of epoxides via alcohols to give the corresponding ß-alkoxy alcohols **23** up to a 99% yield after 0.5 to 20 h at room temperature (Scheme 15) [63]. The reactivity of the catalyst depended on the counterion, with FcBF_4_ showing better performance than FcPF_6_. The orientation of the ring opening depended on the substituent on the epoxide ring system. In the case of an aromatic substituent, the internal carbon was attacked by the alcohol nucleophile, and in aliphatic epoxides, the terminal carbon was attacked.

**Scheme 16:**
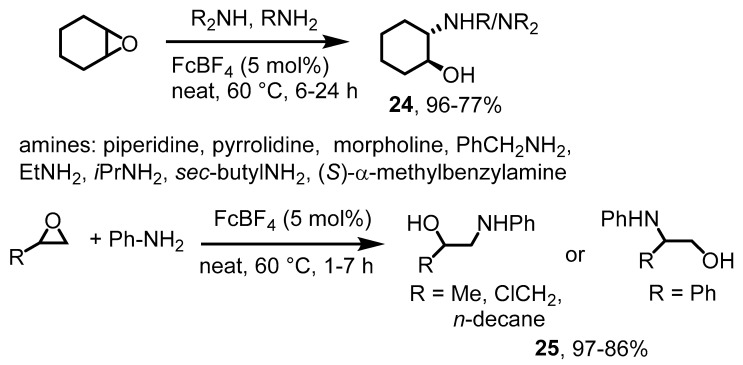
Aminolysis of Epoxides [64].

The same authors reported the ring opening of epoxides via amine nucleophiles (Scheme 16) [64]. Under the same solvent-free conditions as in Scheme 15, ß-amino alcohols **24** or **25** were in yields of up to 99% after 1 to 24 h at room temperature. The orientation of the ring opening was identical to that in the alcoholysis reaction in Scheme 15.

**Scheme 17:**
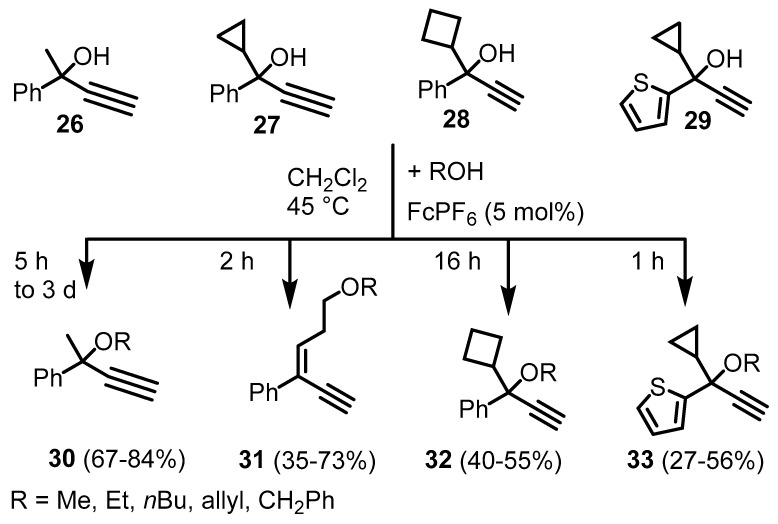
Ferrocenium-catalyzed propargylic substitution reactions [65,66].

In the author of this review article’s own laboratory, ferrocenium cations were employed in propargylic substitution reactions (Scheme 17) [65,66]. When the propargylic alcohols **26**–**29** reacted with nearly stoichiometric amounts of alcohol nucleophiles in the presence of catalytic quantities of FcPF_6_, the resulting substitution products **30**–**33** were obtained in yields ranging from 84% to 35%. The reaction rate was influenced by the substituents on the carbon atom of the propargylic alcohol bearing the OH group, with more carbocation-stabilizing substituents accelerating the reaction. For instance, the thiophenyl-substituted, electron-rich propargylic alcohol **29** reacted much faster than the methyl-substituted propargylic alcohol **26**. This observation suggests a cationic mechanism, where the ferrocenium cation facilitates the formation of a propargylic carbocation intermediate that reacts with the alcohol nucleophile to yield the substitution product. Notably, the cyclopropyl-substituted propargylic alcohol underwent ring opening, producing the more structurally complex ene-yne substrate **31**.

As can be seen in the examples above, the ferrocenium cation had been employed as a Lewis acid catalyst in a number of transformations. In all the cases, weakly coordinating counterions were employed, which did not compete as strongly with the substrates for coordination to the cationic iron center. In the reactions where nucleophilic substrates were employed (like the alcoholysis and aminolysis reactions in Scheme 15 and Scheme 16), a non-nucleophilic counterion was essential to avoid competition with the electrophile. An important factor was the solubility of the ferrocene or the ferrocenium cation. Solubility depends on factors such as the counterion and the solvent. Ferrocenium salts are soluble in a wide range of polar organic solvents, including CH_2_Cl_2_ and THF, with ferrocenium tetrafluoroborate having a higher solubility in CH_2_Cl_2_ than ferrocenium hexafluorophosphate but may need some heat to go into the solution [64]. The Lewis acidity could be tuned through electron-withdrawing substituents on the cyclopentadienyl ring systems. This strategy has barely been employed in synthetic organic applications and may open access to more Lewis acidic catalysts with increased reactivity. 

## 5. Ferrocenium-Catalyzed Oligomerization and Polymerization Reactions

Iron-based polymerization catalysts are well established [67], with reports highlighting the use of ferrocenium-based catalysts early on [68]. These ferrocenium catalyzed polymerization reactions can be broadly classified into two categories: radical or cationic initiator catalysis and cationic or radical photopolymerization catalysis [69]. Radical polymerizations can proceed very rapidly, and some do not need stimulating light irradiation. Photopolymerization, which involves curing liquid resin into solid material using UV radiation, is a subject of extensive research and has numerous applications in 3D-printing technology [70]. There is a trend toward using visible LED irradiation for curing [71]. Ferrocenium-based cationic polymerization catalysts have been known for quite a while, most notably the [FeCp(Ar)]^+^PF_6_ photopolymerizaton catalyst system (Ar = benzene, *p*-cymene, toluene and related aromatic rings), whose derivatives have been researched extensively in the past decade [69,72]. Most of these examples are mixed ligand sandwich complexes, where the iron center is coordinated to a Cp ring and an aryl group. Recent examples of ferrocene or ferrocenium-based polymerization catalysts are compiled below. 

**Scheme 18:**
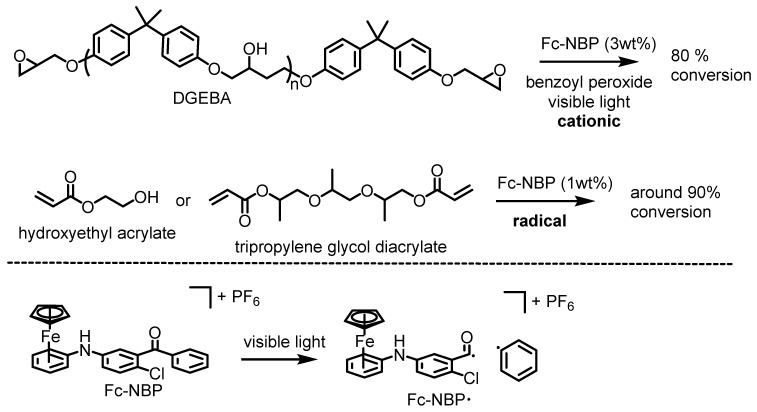
Epoxide polymerizations by a hybrid catalyst through cationic or radical processes [73].

An earlier example of photopolymerization employing a cationic aryl cyclopentadienyl iron polymerization catalyst of the general formula [FeCp(Ar)]^+^PF_6_ was provided by Wang [73]. The cationic complex [Fe(Cp)(η^6^-3-benzoyl-4-chlorodiphenylamine)]PF_6_ (Fc-NBP, Scheme 18) was employed in the photopolymerization of the diglycidyl ether of the bisphenol-A epoxy (DGEBA) to reach conversions up to 80%. The polymerization could be accelerated by an addition of benzoyl peroxide. Under visible light irradiation from a halogen lamp, the catalyst Fc-NBP demonstrated catalytic activity in the radical polymerization of acrylates, achieving conversions of up to 90%. The radical polymerization was not accelerated by the addition of benzoyl peroxide, showing that the radical initiation took mainly place on the ferrocenium catalyst Fc-BNP, by producing the radical Fc-NBP (Scheme 18). 

**Scheme 19:**
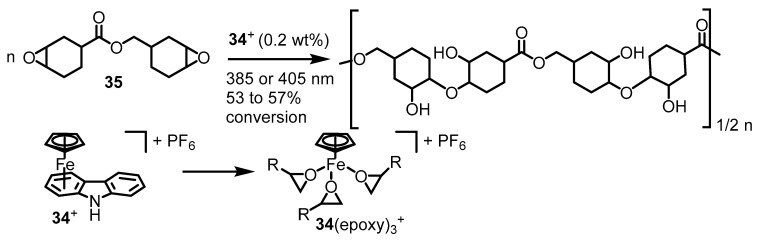
Epoxide ring opening polymerization with a cationic aryl cyclopentadienyl carbazole iron complex [75].

Xiao and Lalevée, revisiting earlier work [72,74], investigated a cationic aryl cyclopentadienyl carbazole iron complex **34**^+^PF_6_ as a cationic photoinitiator in the near-UV and visible LED region for the cationic ring-opening polymerization of epoxide **35** (Scheme 19) [75]. Epoxide conversions of 55–66% were obtained after 800 s of irradiation at 385 or 405 nm. Complex **34**^+^PF_6_ is active even without photoinitiation. The authors assumed that the neutral carbazole ligand was replaced by epoxides from species **34**(epoxy)_3_^+^, where the epoxides exhibited increased electrophilicity, facilitating nucleophilic attacks via another epoxide unit. 

**Scheme 20:**
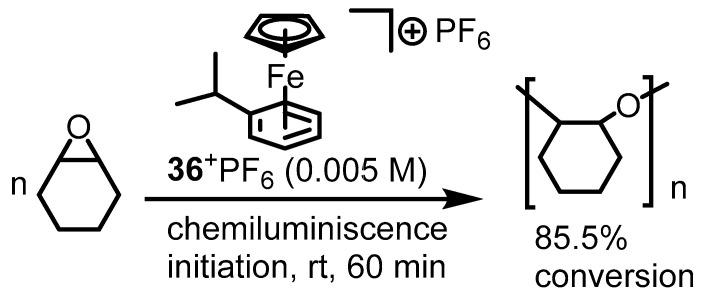
Chemiluminiscence-induced epoxide polymerization [76].

Chemiluminiscence, i.e., the spontaneous emission of light from an exited state of an intermediate, provides another method to provide the light required for photopolymerization. The key advantage of chemiluminescence is that it eliminates the need for photochemical equipment. Li provided an example of the cationic photopolymerization of cyclohexene oxide, where the **36**^+^PF_6_ catalyst was activated by chemiluminescence (Scheme 20) [76]. The polymer was obtained with 85.5% conversion. The monomers *N*-vinylcarbazole, *n*-butyl vinyl ether, and oxetane could be polymerized in the same way, in conversions ranging from 80 to 99.8%. The chemiluminescence was generated via the oxidative cleavage of bis(2,4,6-trichlorophenyl)oxalate (TCPO) by hydrogen peroxide, which generated a chemiexcited dioxetanedione intermediate, which decomposed to release CO_2_ and transferred energy to 9,10-diphenylanthracene (DPA), resulting in bright blue fluorescence at around 430 nm. The **36**^+^PF_6_ catalyst was similarly used to cure an epoxide monomer under LCD light for a 3D-printing application [77].

The aforementioned examples were examples of photopolymerization. Ferrocenium cations can also induce radical polymerizations.

**Scheme 21:**
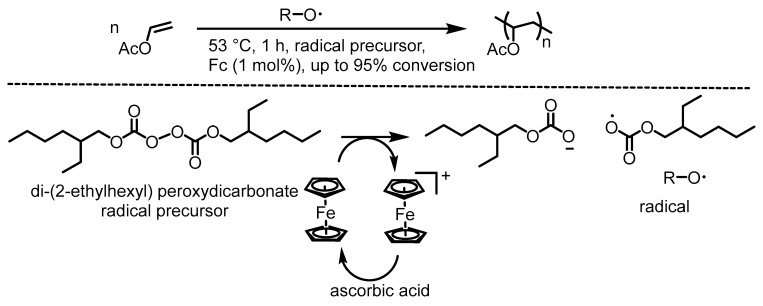
Radical polymerization with ferrocene in catalytic amounts [78].

Mortreux, Champouret, and Visseaux employed ferrocene in the polymerization of vinyl acetate under suspension conditions (Scheme 21) [78]. In this approach, ferrocene served as initiator for the reductive O-O bond cleavage in di-(2-ethylhexyl) peroxydicarbonate to obtain a radical and a carbonate anion. The radical initiated the polymerization of the vinyl acetate to the polyvinyl acetate in up to 95% conversion (53 °C, 2 h). Ascorbic acid was added to the reaction mixture to reduce the ferrocenium species back to ferrocene to maintain the radical formation process. Through the addition of ascorbic acid, the amount of ferrocene could be reduced to catalytic amounts, reducing the metal content of the final polymer product. Vinyl chloride was polymerized in a similar manner using a ferrocene/ascorbic acid palmitate ester activator, demonstrating the need to tailor the activator to the specific monomer used in the polymerization process.

**Scheme 22:**
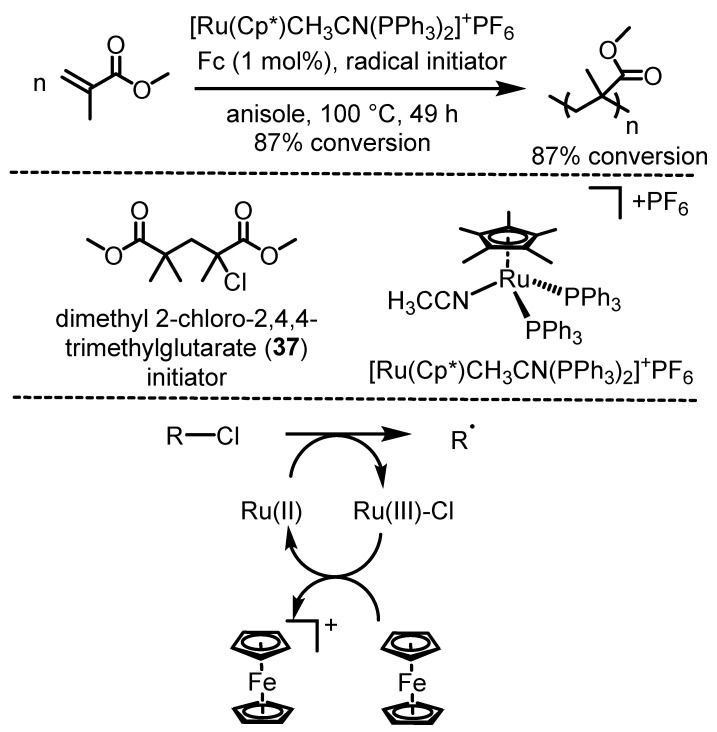
Living radical polymerization of methyl methacrylate [79].

In a related approach, ferrocene was utilized to assist in radical formation as a polymerization initiator through the ruthenium complex [Ru(Cp*)CH_3_CN(PPh_3_)_2_]^+^PF_6_ [79]. This cationic ruthenium(II) complex was employed in reductively converting a dormant chloride (dimethyl 2-chloro-2,4,4-trimethylglutarate initiator, **37**) into a radical initiator (Scheme 22). As in the example above, the ferrocene reduced the Ru(III) back to the Ru(II) species to keep the radical formation alive. The system was employed in the living radical polymerization of methyl methacrylate in 87% conversions after 49 h at 100 °C. The ferrocene itself did not catalyze the polymerization and the polymerization was inhibited in the absence of ferrocene, demonstrating that ferrocene serves as a cocatalyst assisting in the reduction in Ru(III) back to Ru(II). In a related process, the same author reported the FeBr_2_/FeCp*_2_ system in the polymerization of methyl methacrylate in around a 90% conversion in THF at 60 °C [80]. Here, ferrocene as a cocatalyst was less efficient, and the FeBr_2_/ferrocene system resulted in polymerization of up to only about 70% conversion.

The examples above demonstrate that ferrocene and ferrocenium cations can be utilized in polymerization reactions in different ways. Interestingly, the ferrocenium cation itself appeared not to be catalytically active in polymerizations. The cationic species of the general formula [FeCp(Ar)]^+^PF_6_ are catalytically active, but they must be activated, e.g., by irradiation. Here, the neutral, aromatic Ar ligand, which is less firmly bound to the iron center, underwent ligand exchange with the substrate. In other examples, ferrocene or ferrocenium ions play either the role as a radical initiator or as cocatalysts. These systems can be complex and the tailoring of the catalytic system to the monomers is necessary [78]. Still, the employment of iron-based catalysts or cocatalysts can be of interest to the industry.

## 6. Ferrocenium in Redox-Switchable Catalysts

Redox-switchable catalysts are a class of catalyst systems whose activity can be reversibly controlled in situ by changing the oxidation state of their ligands through redox processes [81,82]. The concept involves incorporating a redox-active ligand [83] or a functional group into the architecture of the catalyst, allowing the electron donating or withdrawing ability of the ligand to be modified via oxidation or reduction [84]. These changes in the electronic environment around the metal center can directly influence the reactivity of the catalyst, and potentially, also, its selectivity. Redox-switchable catalysis provide a tool for fine-tuning reactivity in situ, enabling dynamic control over catalytic processes [85].

Ferrocene is an ideal candidate to be incorporated into a redox-switchable catalyst system, because it can easily attach to ligand architectures through the cyclopentadienyl ligands and has a well-behaved Fe(II)/Fe(III) redox couple [86,87]. Related systems were initially described by Wrighton for a cobaltocene system [88]. An early ferrocene-based example was provided by Mirkin, where a rhodium complex with ferrocene-containing ligands was employed in the isomerization of an allyl ether to a vinyl ether [89]. The reaction rate increased when the ferrocene in the ligand was oxidized to the ferrocenium cation. More recent examples are compiled below.

**Scheme 23:**
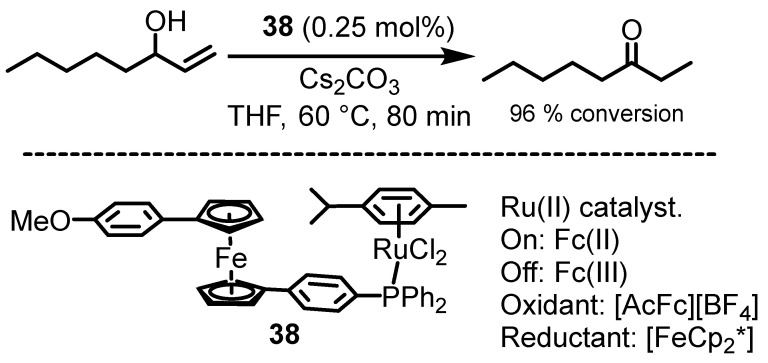
Isomerization reaction with a redox-switchable catalyst [90].

Hey-Hawkins employed a ferrocene-modified phosphine ligand to be coordinated to a [RuCl_2_(cymene)] unit (**38** in Scheme 23) [90]. The complex was catalytically active in the isomerization of 1-octen-3-ol to 3-octanone. When the ferrocene unit in **38** was oxidized to the corresponding Fe(III) species, the catalytic activity was notably slowed; catalytic activity was regained upon reduction back to the Fe(II) ferrocene species. Catalytic activity can be controlled through a redox process at the ferrocene. The catalyst system was incorporated into a dendrimer system, which increased the overall catalytic activity, but the redox switch remained operative. The decrease in activity was not attributed to changes in the solubility, as the reaction mixture stayed homogeneous.

**Scheme 24:**
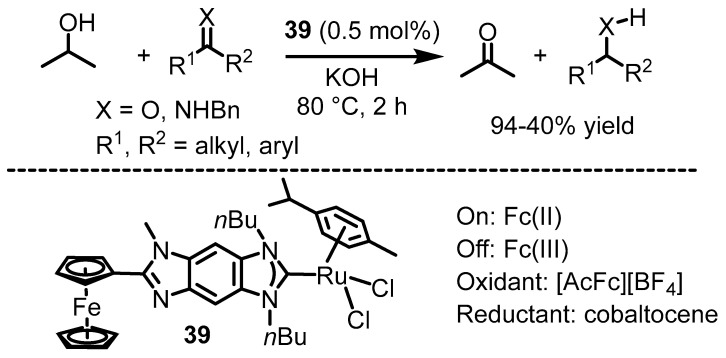
Transfer-hydrogenation catalyzed by a redox-switch [91].

A related system was described by Poyatos and Peris (Scheme 24) [91]. A [RuCl_2_(cymene)L] complex (**39**) containing a ferrocenyl–benzo-fused imidazolylidene ligand was employed in the transfer hydrogenation of ketones and imines, using isopropyl alcohol as the hydrogen source. Again, the neutral complex with a ferrocenyl substituent showed high activity, which was significantly reduced when the ferrocenyl Fe(II) unit was oxidized to the ferrocenium Fe(III) species. The catalytic activity was restored upon reduction in the Fe(III) back to Fe(II).

**Scheme 25:**
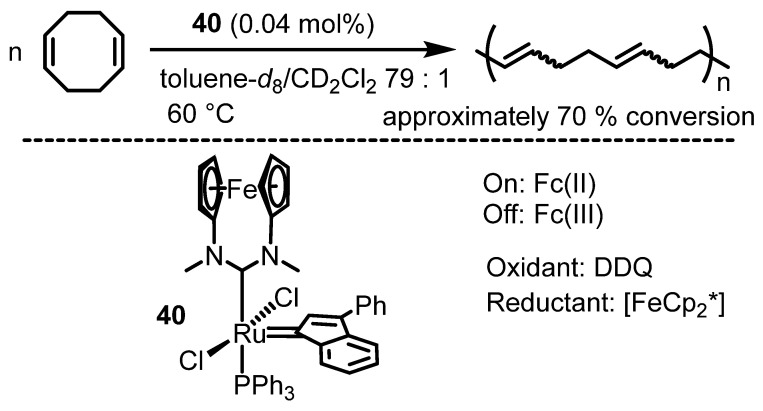
Ring-opening metathesis polymerization [92].

Bielawski presented ruthenium carbene complex [(L)(PPh_3_)Cl_2_Ru=CR_2_] **40**, where L was a *N*,*N*′-dimethyldiaminocarbene[3]ferrocenophane ligand (Scheme 25) [92]. The complex showed catalytic activity in the ring-opening metathesis polymerization of 1.5-cyclooctadiene. The oxidation of the ferrocenyl unit in the ligand with 2,3-dichloro-5,6-dicyanoquinone (DDQ) reduced the catalytic activity of the system by an order of magnitude, which was restored when the ferrocenium was reduced back to the ferrocenyl species using the more electropositive FeCp*_2_. No precipitation was observed during the oxidation, demonstrating that the decrease in catalytic activity could not be attributed to decreased solubility of the oxidized species.

**Scheme 26:**
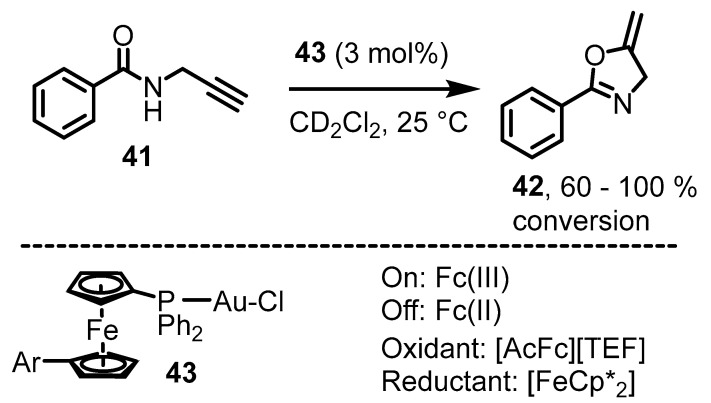
Ring-closing isomerization [93].

A redox-switchable gold complex was applied by Hey-Hawkins in the 5-exo-dig ring-closing isomerization of *N*-(2-propyn-1-yl)benzamide **41** to obtain cyclic products **42** (Scheme 26) [93]. In this study, homotrinuclear gold(I) complexes bearing tris(ferrocenyl)arene-based tris-phosphanes were synthesized and characterized (**43** in Scheme 26, simplified). The complexes showed catalytic activity in the cycloisomerization reaction; the activity increased significantly when the ferrocenyl units were oxidized to ferrocenium utilizing acetyl ferrocenium TEF, [AcFc][TEF] (TEF = tetrakis(perfluoro-*t*butoxy)aluminate). Due to the trinuclearity of the catalyst, the catalytic activity increased stepwise after the addition of one, two, or three equivalents of the oxidant. The addition of a reductant slowed the reaction, and the catalyst system was, thus, redoxswitchable. The authors speculated that the oxidation of the ferrocenyl ligand increased the Lewis acidity of the Au(I) catalytic centers, leading to increased catalytic activity.

**Scheme 27:**
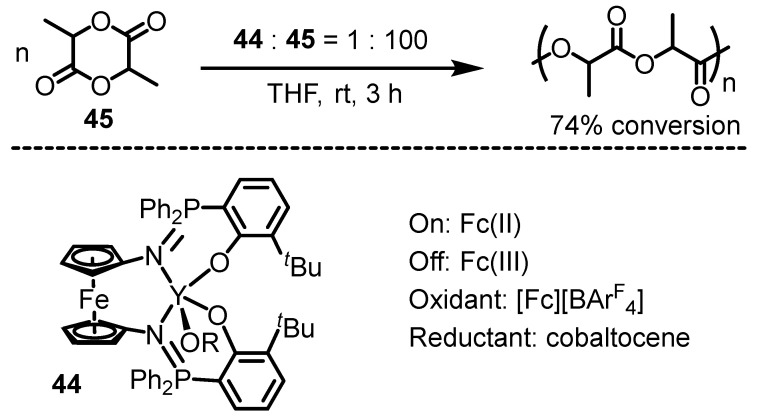
Ring-opening polymerization of L-lactide (**45**) [94,95].

An yttrium alkoxide phosphine complex (**44**) with a ferrocene-based ligand was reported by Diaconescu to exhibit catalytic activity in the ring-opening polymerization of L-lactide (**45**, Scheme 27) at room temperature [94,95]. Conversions of 74% were obtained. Upon in situ oxidation with [Fc][BAr^F^] (BAr^F^ = tetrakis[3,5-bis(trifluoromethyl)phenyl]borate), the catalytic activity decreased, but was restored when the complex was reduced with cobaltocene (CoCp_2_). Mössbauer spectroscopy confirmed that the oxidation occurred at the ferrocenium backbone, rather than at the yttrium center. In contrast, an analogous indium catalyst (M = In instead of Y in **44**) was barely active in the polymerization of trimethylene carbonate, but its activity increased upon oxidation with [Fc][BAr^F^]. The opposite behavior was observed for the yttrium complex **44**, which was more active than the polymerization of trimethylene carbonate in its reduced form. This example illustrates the intricate relationship between the oxidation state of the ferrocenyl substituent and its impact on catalytic activity, which appeared to be metal-dependent.

**Scheme 28:**
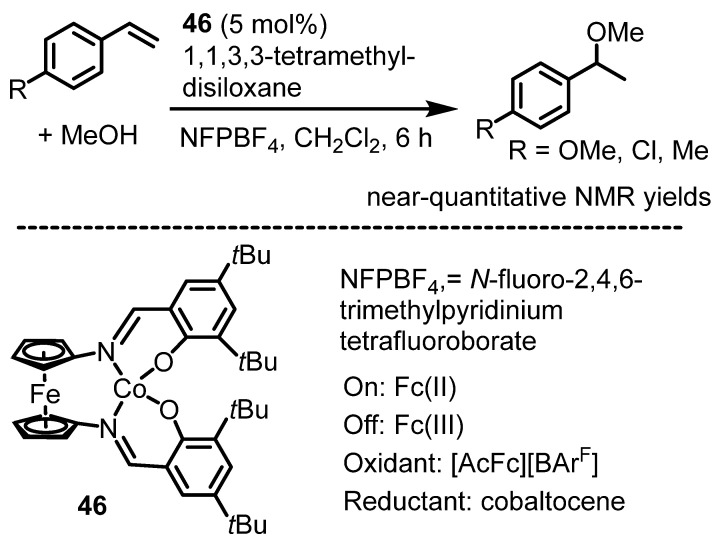
Hydroalkoxylation of styrene derivatives [96].

Diaconescu also reported the cobalt complex of the salfen ligand, 1,1′-di(2,4-di-*tert*-butyl-6-salicylimine)ferrocene (**46** in Scheme 28) [96]. This complex underwent two ligand-centered oxidations. The neutral form exhibited catalytic activity in the hydroalkoxylation of the styrene derivatives in near-quantitative yields within hours. However, the in situ oxidized species, generated with [AcFc][BAr^F^], showed no catalytic activity, which was restored upon reduction with cobaltocene.

**Scheme 29:**
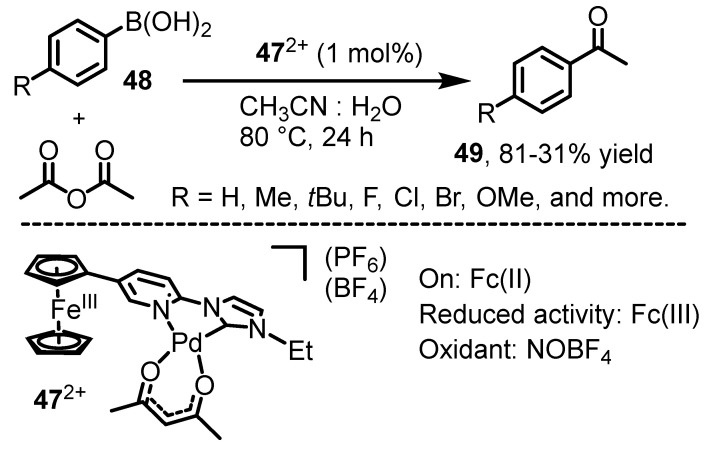
Cross coupling of arylboronic acids and acetic anhydride [97].

Saha reported the use of the ferrocenium-substituted dicationic complex **47**^2+^ as a catalyst for the cross-coupling of aryl boronic acids (**48**) with acetic anhydride to produce the corresponding aryl ketones (**49**, Scheme 29) [97]. The reactions, conducted at 80 °C for 24 h, yielded products with yields ranging from 31% to 100%. The dicationic complex **47**^2+^ was prepared by oxidizing the ferrocene moiety in **47**^+^ to the ferrocenium cation using NOBF_4_. Notably, the mono-cationic complex **47**^+^ outperformed **47**^2+^, affording yields approximately 20% higher. While the oxidation of the catalyst did not completely inhibit catalytic activity, it did lead to a noticeable reduction in activity.

The studies summarized above have demonstrated that the oxidation of the ferrocenyl group on ligands can either enhance or diminish catalytic activity depending on the metal center and reaction pathway, offering a tunable approach to catalysis. Redox-switchable catalysts can improve selectivity, and reduce the energy required for a catalytic transformation. The research field is lively, and further advances can be expected.

However, significant gaps remain in understanding the principles governing the effect of the redox switch. The correlation between the ferrocenyl oxidation state and catalytic behavior is often system-specific, and the mechanistic pictures of these redox-induced transformations are not yet fully understood. The activity seems to be metal-dependent. For example, the isomerization of the allyl alcohol by a ruthenium complex in Scheme 23 is more efficient when the ferrocenyl species is present in its reduced form [90], whereas the isomerization of an allyl ether with a rhodium complex described by Mirkin is more efficient with the ligand of the catalytic species being present in its oxidized form [89]. The yttrium complex **44** (Scheme 27) exhibits greater activity in polymerization reactions when the ligand is in its reduced form. In contrast, the same complex with an indium center shows enhanced activity when the ligand is in its oxidized form [94]. The exact reason for this behavior remains unclear and requires further investigation; DFT calculations have been suggested for that purpose [94]. Improving the durability of these systems under catalytic conditions also poses challenges because sometimes the initial rate acceleration leveling is off over time [89]. Further research is needed to generalize the principle and expand the applicability of redox-switchable catalysts based on the ferrocene/ferrocenium couple to a wider range of chemical transformations.

## 7. Conclusions and Perspective

Ferrocenium-catalyzed reactions offer promising opportunities in both organic synthesis and polymer chemistry, particularly due to the unique Lewis acid and redox properties of the ferrocenium cation. However, one significant limitation is the relatively low stability of ferrocenium cations in solution, where they are prone to oxidative decomposition [98,99]. This instability can limit their catalytic efficiency, requiring the careful control of reaction conditions such as temperature and atmosphere (e.g., inert gas environments). The solvent choice can also be crucial; it has been demonstrated that the decomposition slowed [98] and the catalytic activity of the ferrocenium cations increased [57] when a perfluorinated solvent was used. Despite this, ferrocenium cations have shown great potential in promoting cationic and radical polymerizations, as well as in facilitating substitutions and related reactions by creating carbocation intermediates or by polarizing substrates. Additionally, their tunable redox behavior allows for catalytic versatility, enabling ferrocenium to act as both an oxidant and an initiator under the right conditions. Further research into stabilizing ferrocenium cations or designing more robust ferrocenium derivatives could open new reaction pathways and enhance their catalytic utility in a wider range of applications.

## Data Availability

Not applicable.
